# Computational Studies of Beta Amyloid (A*β*42) with p75NTR Receptor: A Novel Therapeutic Target in Alzheimer's Disease

**DOI:** 10.1155/2014/736378

**Published:** 2014-11-11

**Authors:** Shine Devarajan, Jeya Sundara Sharmila

**Affiliations:** ^1^Department of Biotechnology and Bioinformatics, Dr. D. Y. Patil University, Navi Mumbai, Maharashtra 400614, India; ^2^Department of Nanoscience and Technology, Tamil Nadu Agricultural University, Coimbatore 641003, India

## Abstract

Alzheimer's disease is a neurodegenerative disorder characterized by the accumulation of beta amyloid plaques (A*β*) which can induce neurite degeneration and progressive dementia. It has been identified that neuronal apoptosis is induced by binding of A*β*42 to pan neurotrophin receptor (p75NTR) and gave the possibility that beta amyloid oligomer is a ligand for p75NTR. However, the atomic contact point responsible for molecular interactions and conformational changes of the protein upon binding was not studied in detail. In view of this, we conducted a molecular docking and simulation study to investigate the binding behaviour of A*β*42 monomer with p75NTR ectodomain. Furthermore, we proposed a p75NTR-ectodomain-A*β*42 complex model. Our data revealed that, A*β*42 specifically recognizes CRD1 and CRD2 domains of the receptor and formed a “cap” like structure at the N-terminal of receptor which is stabilized by a network of hydrogen bonds. These findings are supported by molecular dynamics simulation that A*β*42 showed distinct structural alterations at N- and C-terminal regions due to the influence of the receptor binding site. Overall, the present study gives more structural insight on the molecular interactions of beta amyloid protein involved in the activation of p75NTR receptor.

## 1. Introduction 

Alzheimer's disease (AD) is the most common form of dementia and is a neurodegenerative disorder characterised by the excess production of amyloidogenic beta amyloid proteins. These further lead to various conditions include mood disorders, cognitive dysfunction, and neuronal death. The hallmark of the disease in the brain is the accumulation of soluble amyloid beta protein (A*β*) which is produced by cleavage of the amyloid precursor protein [1]. This amyloid hypothesis is because of the A*β* oligomers rather than amyloid deposits that lead to the neurotoxicity associated with this condition [2–4]. Among the two variants of amyloid beta protein, A*β*42 aggregates much faster and is more amyloidogenic than A*β*40 [5]. However, the pathogenesis of AD is not fully understood, and many disease modifying pathways and macromolecules that are obviously involved need to be studied. Another observation indicated in Alzheimer's disease is the degeneration of the cholinergic basal forebrain neurons, which expressed elevated levels of pan neurotrophin receptor (p75NTR) in the adult brain [6].

When p75NTR is activated by nerve growth factor (NGF) in cells that do not also express the NGF-specific specific receptor p140trkA, p75NTR activation leads to cell death [7, 8]. Further study also confirmed in PC12 cells that like neurons undergo cell death when exposed to A*β* so the expression of p75NTR receptor is required for A*β* to kill these cells [9]. It has been previously reported that both the soluble and aggregated form of A*β* induced apoptosis can be mediated through its binding to p75NTR receptor and given that A*β* is a ligand for this receptor [10]. Moreover, there is strong evidence which indicated that p75NTR is required for oligomeric A*β*42 mediated neuronal death* in vitro* and* in vivo*, and this strengthens the role of receptor in the aetiology of Alzheimer's disease [11]. There are experimental evidences which demonstrated that A*β* binds not only with p75NTR monomer receptor but also with P75 trimers on the surface of neurons which induced receptor activation [12]. The above study strongly suggested that A*β*/p75NTR interaction is related to neuronal loss in AD. Another experimental study further established the role of targeting p75NTR in AD using fluorescence resonance energy transfer- (FRET-) based technology to investigate oligomeric A*β* interactions with the extracellular domain of p75NTR and concluded that p75NTR is required for A*β* induced deleterious signalling and neurodegeneration [13].

However, no further details are available to explore the binding domain between these proteins and to trace the conformational changes which trigger p75NTR activation that lead to neuronal death. The binding mode between A*β*42 and p75NTR ectodomain has remained unknown. Furthermore, there is no molecular interaction study performed computationally to establish the conformational changes and reveal the binding domains of A*β*42 and ectodomain of p75NTR receptor. In view of this, we performed a molecular docking study to see the contact points and domains involved in the proteins. Secondly, a molecular dynamics simulation was performed to get more insight on the conformational changes established upon binding of A*β*42 monomer to the receptor. We observe that A*β*42 monomer shows high binding affinity to the extracellular domain of the receptor which is stabilized by hydrogen bonds in cysteine rich domains of p75NTR and various amino acid residues between N- and C-terminals of A*β*42. The dynamics of the complex is studied by performing a molecular simulation (MD) and explored the conformational changes of the proteins. The simulated model shows partial unfolded beta amyloid (A*β*42) structure due to the influence of the receptor binding site. We study the model by analyzing the RMSF to see the stability of backbone and atomic fluctuation of the complex.

Neurite degeneration is the critical event of Alzheimer's disease and the major contributor of dementia and A*β*42 is an important factor that leads to this process. However, p75NTR does not always regulate A*β* induced apoptosis; it is also proposed to exert neuroprotective effect against A*β* toxicity in neuron cells [14, 15]. The present study gives a detailed account on how this short peptide recognized and alters its conformation through the extracellular domain of p75NTR. Indeed, this mechanism will shed a light on the p75NTR induced signalling cascade for neuronal death.

## 2. Materials and Methods

### 2.1. Molecular Docking

**Figure 1:**
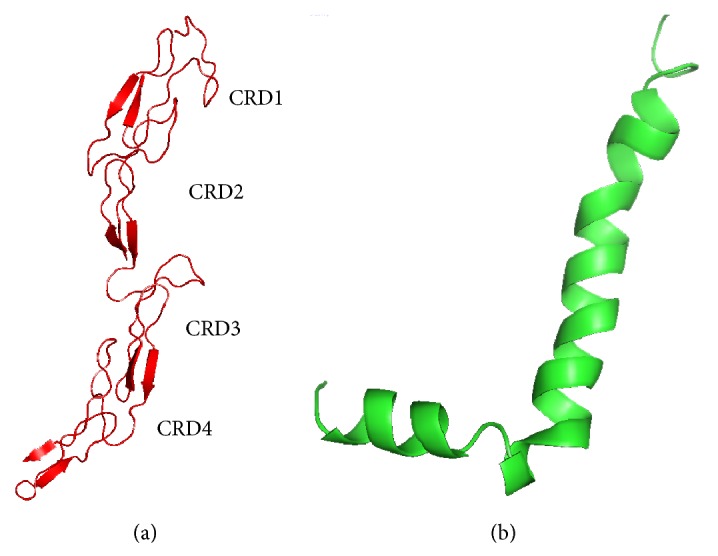
(a) Secondary structure of p75NTR ectodomain protein where four cysteine rich domains (CRD1, CRD2, CRD3, and CRD4) are shown. (b) A*β*42 protein is represented in cartoon ribbon.

The amyloid beta protein or A*β*42 (1–42 residues) was obtained from the first model of the protein data bank entry 1IYT [16]. Since it is an NMR model, the structure was checked for any steric clashes between amino acids by spdbv software [17]. The crystal structure of p75NTR receptor was taken from the PDB entry 3BUK [18], which was a symmetrical 2 : 2 complex between p75NTR and neurotrophins. In the present study, we have used a single monomer from the above p75NTR ectodomain structure (Figure 1(a)) and A*β*42 oligomer (Figure 1(b)).

Cluspro 2.0 [19–22] protein-protein docking algorithm which worked in three main steps was used to study the molecular interactions and binding. In first step, it runs PIPER, based on a Fast Fourier Transform (FFT) docking method. Secondly, it used a clustering approach for the identification of near native conformations and discards the unstable clusters. Finally, a short Monte Carlo simulation was applied to judge the stability of these clusters and further refined. We have selected our best model based on cluster size and the parameters generated by balanced, electrostatic, hydrophobic, and van der Waals + electrostatic.

### 2.2. Molecular Dynamics

In our study, we performed molecular dynamics simulation (MD) of A*β*42 and p75NTR complex using Gromacs 4.0.5 package [23–25]. The proteins were parameterized with GROMOS96 53A6 force field [25] and solvated using SPC water model [26] in a dodecahedron box of dimension 96 Å × 96 Å × 45 Å. The system was (charge-26.0) neutralized with required Na^+^ counter ions. Then, it was treated for energy minimization by steepest descent method and the system converged in 1723 steps. The complex was then subjected to NPT equilibration for a period of 1 ns where the number of particles, pressure, and temperature kept as constant parameters. Berendsen temperature coupling [26] was set for 300 K and applied 1 atm pressure by Parrinello-Rahman algorithm [27]. The linear constraint solver (LINCS) algorithm [28] was provided to constrain all the bonds in the complex system. Long range electrostatics parameter was measured using the Particle Mesh-Ewald (PME) method [29] with a Fourier grid spacing of 0.16 Å and a spline order of 4. The final simulation was carried out using the leap-frog integrator for a period of 2 ns with a time step of 2 fs.

## 3. Results and Discussion

In the present study, we use 42-amino acid length A*β* protein and study its binding site with p75NTR receptor. A*β*42 protein monomer is used throughout the study with p75NTR ectodomain monomer. In regard to the computational investigations, we perform molecular docking followed by a dynamics simulation of A*β*42 protein with p75NTR ectodomain monomer to study if any conformational changes and molecular specificity established between these two proteins.

### 3.1. Molecular Interactions between A*β*42 and p75NTR Ectodomain Monomer

We perform molecular docking between A*β*42 and p75NTR ectodomain monomer. Cluspro carried out a cluster analysis and identified 28 clusters and the best one had more cluster members and lowest energy compared to other members. We select cluster 1 because it shows lowest energy (−813 kcal/mol) and has desirable nonbonded interactions. The predicted model is quite good in terms of its electrostatic, van der Waals and larger cluster size. A recent study proposed a 2 : 2 Neurotrophin-3 (NT3) and p75NTR symmetrical complex reflects a native state of p75NTR activation at the cell surface through a series of cysteine-rich domains (CRDs) and the interactions were stabilised by number of hydrogen bonds and salt bridges [18]. However, we identify that A*β*42 protein is not oriented parallel to p75NTR, despite the fact that it seems to bind at the N-terminal of the receptor. At the same time, the C-terminal region of A*β*42 binds on the top of p75NTR ectodomain and formed a “cap” like structure. Moreover, the binding site of the receptor for neurotrophin (NT) belongs to cysteine rich conserved domains named CRD1, CRD2, and CRD3. This observation is also supported by experimental findings that nerve growth factor and A*β* binding sites within p75NTR seem to be distinct [30]. In addition to this, it is also proposed that the present contact points and binding behaviour of A*β* within the active site of the receptor will be same as with A*β*40 because p75NTR mediated cell death and activation of signalling cascades triggered by A*β* do not vary with protein size (A*β*1–40/1–42 versus A*β*25–35) [31].

**Figure 2:**
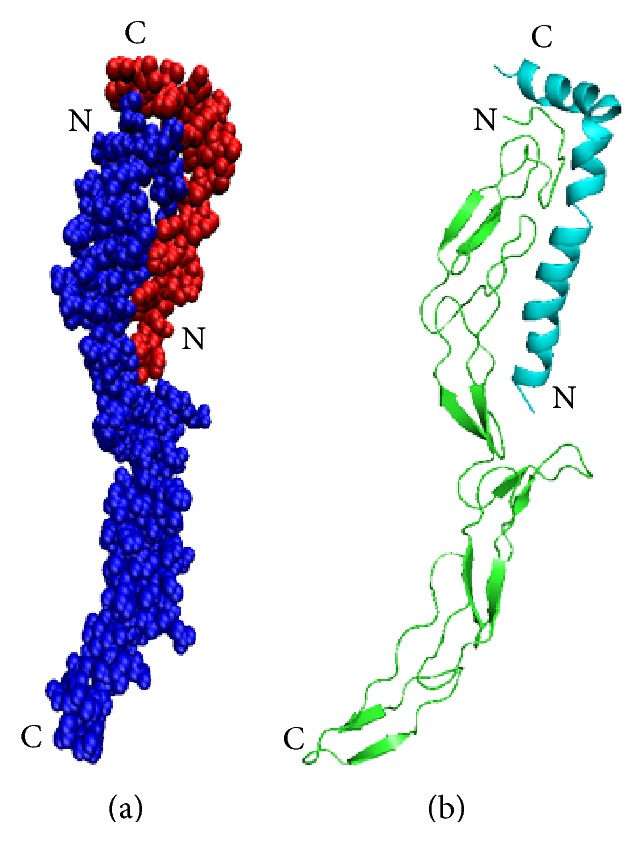
Docked complex of p75NTR ectodomain with A*β*42. The N- and C-terminals of the proteins are labelled. (a) van der Waals surface of the complex (p75NTR in blue and A*β*42 in red). (b) cartoon representation (p75NTR in green and A*β*42 in cyan colour). In (a) & (b), A*β*42 specifically binds and formed a favourable contact points at the N-terminal region of the receptor.

Our docking study reveals a number of hydrogen bond interactions between the ectodomain and beta amyloid for stabilizing the complex. It has been suggested that amino acids within 29–35 regions of A*β* sequence are crucial for the effects mediated by p75NTR [31]. However, in our study, Ile41 and Gly37 of A*β*42 are hydrogen bonded with Thr3 and Ser5 of p75NTR, respectively, and are shown in Table 1 and remaining 10 interactions are observed outside the range mentioned above. In addition to this, cysteine rich domains not directly play a major role with beta amyloid protein except at position Cys79, which belongs to CRD2 of p75NTR. In general, the binding site residues of p75NTR to A*β* are restricted to the amino acids about 2 Å distance nearer to the CRD domains where CRD3 and CRD4 are not directly participating in hydrogen bond interactions. The involvement of His13 which is one of the active site residues of A*β*42 forms a good binding behaviour with Glu53 of the receptor. Moreover, Asp1 at the N-terminal and Lys28 nearer to the C-terminal also showed strong hydrogen bond interaction which indicates the stability of the conformation. The total solvent accessible surface area of the docked complex is about 12014 Å. The docked ligand-receptor complex is shown in (Figure 2).

**Figure 3:**
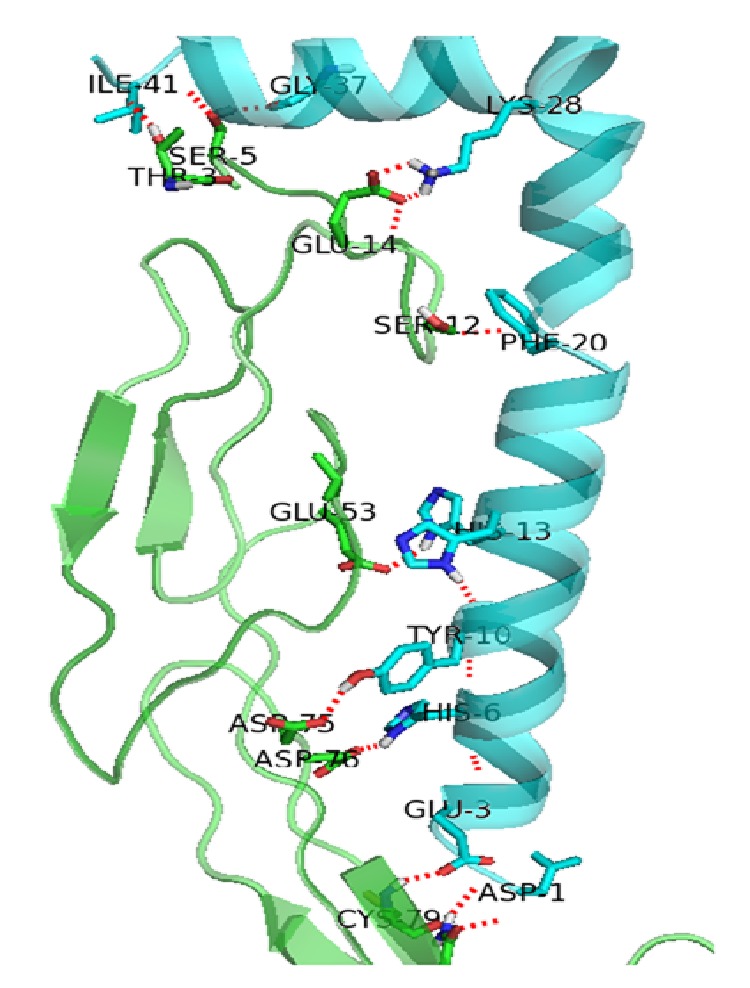
Molecular interaction between p75NTR ectodomain and A*β*42 where the former is shown in green cartoon and the latter in cyan. Hydrogen bonds are shown as red lines between the active site residues where the amino acids involved in the interactions are shown as sticks. CRD3 and CRD4 domain are not shown in the diagram. (Residue numbers are given as per the conventions in Gromacs).

Our molecular interaction study strongly suggests that despite binding with all CRD domains of p75NTR receptor, A*β*42 recognizes the N-terminal region of the receptor and formed a strong hydrogen bond network in the binding site (Figure 3). However, one cysteine amino acid (Cys79) from CRD2 domain participated in hydrogen bond with N-terminal of beta amyloid protein. In addition to this, A*β*42 specifically binds to the topical region of the receptor where the binding was stabilized by a small cleft nearer to CRD2 domain and this supported the “cap” like conformation of A*β*42 ligand.

### 3.2. Molecular Dynamics Study of p75NTR-A*β*42 Complex

**Figure 4:**
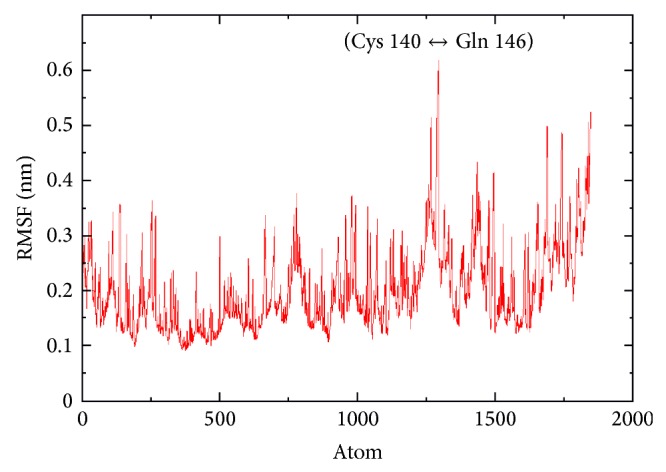
RMSF plot for the system and the large fluctuated atoms belongs to the amino acid ranges which are labelled.

Molecular dynamics (MD) simulation is a popular method for studying the conformational stability of a model. Here, we use Gromacs software to further investigate the quality of the docked complex in detail. The complex obtained from our docking experiment is subjected to MD simulation for 2 ns time scale in an explicit solvent condition. The data collected throughout the trajectory is used to investigate the stability of the secondary structure of the complex by plotting root mean square fluctuation (RMSF) and radius of gyration (Rg).  Figure 4 shows the root mean square fluctuation (RMSF) in order to get the dynamicity of each residue. This indicates that there is a fluctuation between Cys 140 and Gln 146 of p75NTR receptor up to 0.6 nm and this region exhibits more fluctuations compared to the remaining atoms in the complex as shown in (Figure 4). As indicated above, Cys 140 is a part of CRD region of the receptor which further confirms the role of the binding of A*β*42 protein at the respected region.

**Figure 5:**
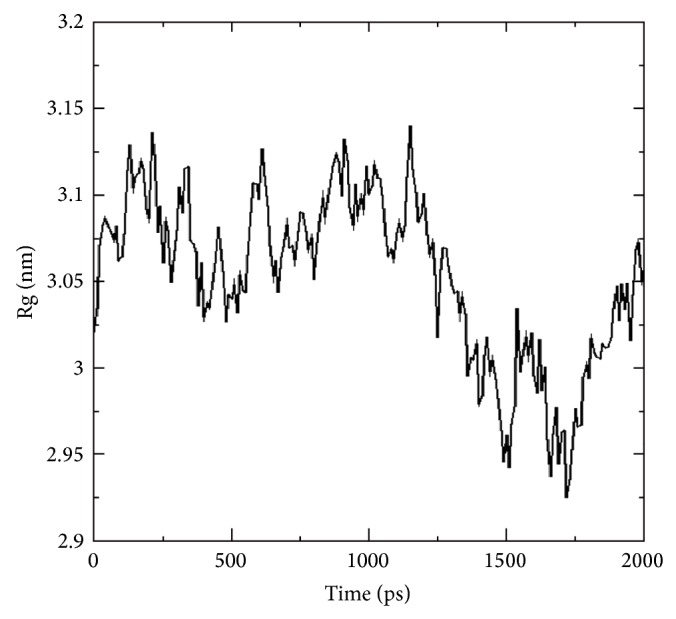
Radius of gyration plot for p75NTR-A*β*42 complex.

Radius of gyration (Rg) of a protein is a measure of its compactness. This parameter gives stability and firmness of the system and tends to change over time due to protein folded-unfolded states [32]. If a protein unfolds, its Rg will change over time. The mean Rg shown in Figure 5 seems to be a decline during 1.75 ns (1700 ps) which indicates the conformational changes of A*β*42 due to structural flexibility upon binding.

#### 3.2.1. Conformational Changes of the Proteins

**Figure 6:**
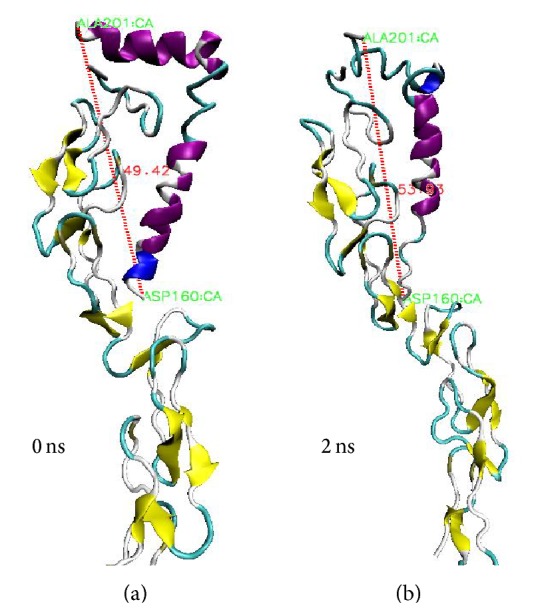
Conformations of p75NTR-A*β*42 complex before and after simulation whereas the backbone p75NTR ectodomain is shown in yellow while A*β*42 in magenta and blue. Bond length between N & C terminus of A*β*42 is represented as red dotted line. (a) Conformation at 0 ns and (b) after 2 ns.

We observed conformational changes especially at the N- and C-terminal regions of beta amyloid protein upon binding to the receptor. The folded *α* helical structure tends to unwind during our short MD simulation which gives the impression that the residues nearer to the CRD2 domain of p75NTR play a significant role in this process shown in Figure 6. There is no structural transition of A*β*42 which occurs between amino acids Asp7 and Phe20. However, the distance between N- and C-terminal regions shows some light on the conformational alterations in A*β*42 structure. In our simulation results, structure at 0 ns gave 49.42 Å distance between the two terminals whereas it showed 53.93 Å after 2 ns. This indicated that A*β*42 is more prone to a coiled form during this duration which provides a scope for further investigating the p75NTR-A*β*42 complex. There are no significant structural changes occurring in p75NTR receptor but the ligand binding site of the receptor (CRD1 & CRD2) shows backbone rearrangements in the complex form which is shown in Figure 6(b).

**Figure 7:**
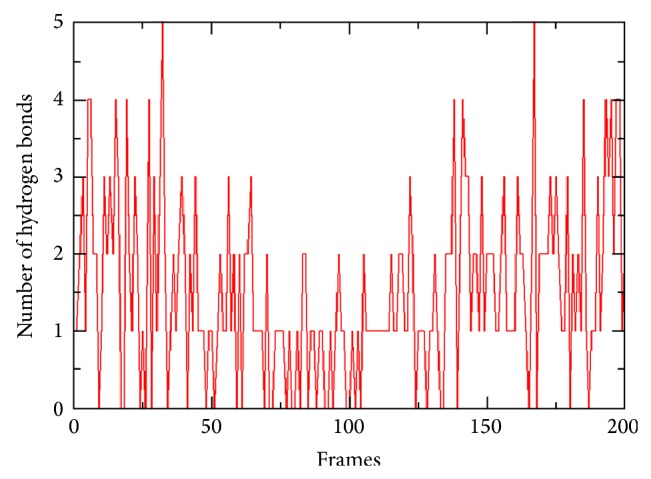
Hydrogen bonds between p75NTR and A*β*42 shown in all the frames.

We observed a number of intra- and inter hydrogen bonds stabilizing the complex throughout the simulation which is shown in Figure 7. Despite the structural alteration of beta amyloid protein in the receptor binding site, the conformation of docked complex is well stabilised by this interactions. More hydrogen bonds are formed nearer to the starting conformation and almost at the end of the simulation. All these findings shed a light on how the binding of A*β* which triggers the process of p75NTR induced death signalling cascade in neurons. Despite the fact that the signalling pathways are complex in the nature, it also needs to explore the conformational changes of the intracellular “death domain” and “chopper domain” upon the binding of A*β*42. Because p75NTR is an integral membrane protein so a detailed molecular dynamics simulation considering the extra- and intracellular domains of p75NTR could give more avenues to study the apoptosis and for therapeutic interventions in Alzheimer's disease.

## 4. Conclusion

The pan neurotrophin receptor (p75NTR) is best known for mediating neural cell death and acts as a target for the treatment of neurodegenerative disease. The study is focused on the binding of A*β* to the ectodomain of p75NTR receptor, which induces apoptosis in nerve cells and activation of signalling cascade triggered by A*β*. Molecular docking and simulation techniques are used to investigate the binding and conformational changes between p75NTR ectodomain and A*β*42 which act as a ligand for p75NTR. This was supported by a number of experimental evidences [10, 12, 13] and we suggest that A*β*42 shows strong binding behaviour to the receptor and the complex is stabilized by a network of hydrogen bond interactions. The proposed molecular model of the ectodomain of p75NTR-A*β*42 complex will give more structural insight as well as the binding pattern for further investigations to other endodomains of the receptor. However, biological assays and various biophysical methods such as X-ray diffraction could be used to further validate the results. Thus, our findings will be useful to analyze the intracellular signalling events mediated through various domains which lead to apoptosis. Furthermore, p75NTR is not only a good therapeutic target but a crucial factor between neuronal survival and cell death in Alzheimer's disease.

## Figures and Tables

**Table 1:** Hydrogen bond interactions from the best docked complex of A*β*42-p75NTR ectodomain based on ClusPro score.

p75NTR residues	Atoms involved	A*β*42 residues	Atoms involved	Distance (Å)
Thr 3	HG1	Ile 41	O	1.83
Ser 5	HG	Gly 37	O	1.84
Ser 12	H	Phe 20	O	1.95
Val 49	H	His 14	NE2	2.28
Gln 65	HE22	Asp 1	O	1.97
Cys 79	H	Glu 3	OE1	1.97
Gln 65	OE1	Asp 1	H	1.98
Asp 75	OD2	Tyr 10	HH	1.96
Asp 76	OD2	His 6	HD1	2.00
Glu 53	OE1	His 13	HD1	2.09
Glu 14	OE1	Lys 28	HZ1	1.78
Glu 14	OE2	Lys 28	HZ3	1.76
